# Flowers for Winter

**Published:** 1864-04

**Authors:** 


**Flowers for Winter.**—Flowers intended for winter blooming need a season of repose, especially tropical plants, such as geranium, fuchsia, etc., whi[illegible] should be allowed rest from growth during the months of July and August, by almost entirely withdrawing the supply of water. Of course the leaves will fall off, but the plants will be fitted to start into fresh and vigorous growth as soon as the water is again supplied. Previous to this, the branches of the fuchsia should be pruned in, and water given sparingly at first, increasing the supply as the young shoots grow.

